# Geographic surveillance of community associated MRSA infections in children using electronic health record data

**DOI:** 10.1186/s12879-019-3682-3

**Published:** 2019-02-18

**Authors:** Lilly Cheng Immergluck, Traci Leong, Kevin Matthews, Khusdeep Malhotra, Trisha Chan Parker, Fatima Ali, Robert C. Jerris, George S. Rust

**Affiliations:** 1 Department of Microbiology/Biochemistry/Immunology, Department of Pediatrics and Clinical Research Center, Morehouse School of Medicine, 720 Westview Drive, SW, Atlanta, GA 30310 USA; 2 Children’s Healthcare of Atlanta, 1405 Clifton Road NE, Atlanta, GA 30322 USA; 3 Rollins School of Public Health, Emory University, 1518 Clifton Rd, Atlanta, GA 30322 USA; 4 Centers for Disease Control and Prevention, Division of Population Health, National Center for Chronic Disease Prevention and Health Promotion, 1600 Clifton Rd, Atlanta, GA 30333 USA; 5 National Center for Primary Care, Morehouse School of Medicine, 720 Westview Drive, SW, Atlanta, GA 30310 USA; 6 Department of Pathology, Emory University, 1364 Clifton Road Northeast, Atlanta, GA 30322 USA; 7 Florida State University College of Medicine, 1115 W. Call St, Tallahassee, FL 32306 USA

**Keywords:** Pediatric, Antibiotic resistant, Spatial analyses, *Staphylococcus aureus* infections, Methicillin resistant *Staphylococcus aureus*

## Abstract

**Background:**

Community- associated methicillin resistant *Staphylococcus aureus* (CA-MRSA) cause serious infections and rates continue to rise worldwide. Use of geocoded electronic health record (EHR) data to prevent spread of disease is limited in health service research. We demonstrate how geocoded EHR and spatial analyses can be used to identify risks for CA-MRSA in children, which are tied to place-based determinants and would not be uncovered using traditional EHR data analyses.

**Methods:**

An epidemiology study was conducted on children from January 1, 2002 through December 31, 2010 who were treated for *Staphylococcus aureus* infections. A generalized estimated equations (GEE) model was developed and crude and adjusted odds ratios were based on *S. aureus* risks. We measured the risk of *S. aureus* as standardized incidence ratios (SIR) calculated within aggregated US 2010 Census tracts called spatially adaptive filters, and then created maps that differentiate the geographic patterns of antibiotic resistant and non-resistant forms of *S. aureus*.

**Results:**

CA-MRSA rates increased at higher rates compared to non-resistant forms, *p* = 0.01. Children with no or public health insurance had higher odds of CA-MRSA infection. Black children were almost 1.5 times as likely as white children to have CA-MRSA infections (aOR 95% CI 1.44,1.75, *p* < 0.0001); this finding persisted at the block group level (*p* < 0.001) along with household crowding (p < 0.001). The youngest category of age (< 4 years) also had increased risk for CA-MRSA (aOR 1.65, 95%CI 1.48, 1.83, p < 0.0001). CA-MRSA encompasses larger areas with higher SIRs compared to non-resistant forms and were found in block groups with higher proportion of blacks (*r* = 0.517, p < 0.001), younger age (*r* = 0.137, p < 0.001), and crowding (*r* = 0.320, p < 0.001).

**Conclusions:**

In the Atlanta MSA, the risk for CA-MRSA is associated with neighborhood-level measures of racial composition, household crowding, and age of children. Neighborhoods which have higher proportion of blacks, household crowding, and children < 4 years of age are at greatest risk. Understanding spatial relationship at a community level and how it relates to risks for antibiotic resistant infections is important to combat the growing numbers and spread of such infections like CA-MRSA.

## Background

The use of geocoded electronic health record (EHR) data for improving health outcomes is in its infancy [1] particularly for infectious diseases such as community-associated methicillin resistant *Staphylococcus aureus* (CA-MRSA) infections [2–4]. This is an important disease to study because it is preventable and the number of children hospitalized with CA-MRSA infections in the United States increased from 6.7 cases per 1000 admissions in 2002 to 21.2 cases in 2007 [5]. We demonstrated how geocoded EHR data in conjunction with a geographic information system (GIS) can be used to enhance the geographic surveillance of CA-MRSA. In doing so, we 1) characterized the communities where children infected by the bacteria *Staphylococcus aureus* (*S. aureus*) resided during the height of the CA-MRSA epidemic in the US [6] (2002–2010); and 2) identified areas at high risk for CA-MRSA where targeted interventions aimed at preventing disease transmission will have potentially the most impact.

Electronic health records are becoming a routine part of patient care. Their primary purpose is to follow the provision of care to individual patients. However, they are also a source of data for public health surveillance that cannot be obtained from reportable disease registries or vital statistics databases. EHR data typically includes basic demographic characteristics of the patient as well as their list of diagnoses, prescriptions, results from laboratory tests, referrals to specialists, and provider characteristics [7]. They also contain residential information about the patient which can be geocoded and linked to data about the patient’s social, physical and built environment, community resources and other data about their community [8]. Use of geocoded EHR for public health is still emerging, but there are a wide range of recent application areas including hospital readmissions for pediatric asthma [9], preventable emergency department visits [10, 11], hospitalizations for common illnesses [12], obesity in children [12], and Type 2 diabetes [8, 13, 14].

Population based studies have shown a higher rate of invasive MRSA infections among blacks and other minorities [15–18] and in densely populated areas with high numbers of pre-school and school aged children [19]. The subset of MRSA infections emerging from the community, or CA-MRSA infections, have been endemic in many urban areas with significantly increased risk for invasive infection among blacks [6, 17, 20]. Risks associated with CA-MRSA disease may also be related to socioeconomic factors [4, 21], and are not well characterized for this community acquired infection [6, 16, 17]. For instance, household crowding has been cited as a risk for CA-MRSA infections, but the impact of neighborhood crowding has not previously been addressed [22, 23].

The goals of this study are to characterize the neighborhoods with the highest rates of CA-MRSA infection in the Atlanta–Sandy Springs–Roswell, GA Metropolitan Statistical Area (the Atlanta MSA), and to analyze the spatial distribution of CA-MRSA compared to the non-antibiotic resistant form of *S. aureus*, also known as community-associated methicillin sensitive *Staphylococcus aureus* (CA-MSSA). There are very few studies to characterize CA-MRSA infections in this region of the US, compared to other parts. This spatial characterization would enable us to identify specific areas at greatest risk for antibiotic resistance and thus, identify those communities most likely to benefit from targeted interventions. Comparing spatial patterns of the nonresistant forms of *S. aureus* to resistant forms would identify clonal spread of specific resistant strains which may be circulating in a community or neighborhood; primary and secondary intervention plans aimed at specific geographic areas could be tailored, based on the risks associated with a particular location. For example, CA-MRSA rates within certain communities [24] have decreased over the last 5 years when community-level prevention measures (e.g., increased hand sanitizers located throughout public areas) were implemented. The importance of achieving these goals would allow public health officials and area primary care providers to combat the spread of CA-MRSA infections through targeted interventions aimed at those communities at highest risk for acquisition and transmission. Such interventions would contribute to the development of health policy guidelines for infection control, effective home and school-based interventions, and identifying but not stigmatizing children who may be at increased risk for CA-MRSA based on individual and area level risk factors.

## Methods

### Overview

CA-MRSA infections in the Atlanta MSA from 2002 to 2010 were examined by applying exploratory spatial analysis in order to identify risk factors and characterize geographic variations of CA-MRSA rates compared to CA-MSSA rates.

### Study design

A retrospective epidemiology study was conducted of children from January 1, 2002 through December 31, 2010 who were treated for *S. aureus* infections at two pediatric hospitals in the Atlanta MSA (Scottish Rite Children’s Hospital and Egleston Children’s Hospital). Children diagnosed with *S. aureus* infection and who were managed in the emergency department (ED), or as an inpatient were included; no data was collected on *S. aureus* carriage or colonization. All had an International Classification of Diseases, Clinical Modification (ICD- 9-CM) code compatible with a staphylococcal infection, a positive *S. aureus* culture, and lived within the 20-counties that comprise the Atlanta–Sandy Springs–Roswell, GA Metropolitan Statistical Area (Metro Atlanta). (The two hospitals consistently provide more than 80% of the pediatric hospitalizations for the Atlanta MSA [25].) This study was approved by Institutional Review Boards of hospitals and affiliated academic institutions.

Each unique child met the case definition if their EHR reported a positive MRSA infection on the first culture during a single hospitalization. MRSA carriage or colonization status is not routinely collected on patients with MRSA infection, so this was not collected for our study. Cases were then categorized as ‘community associated’ or ‘hospital acquired’ infections. Using CDC’s guidelines for case definitions, ‘community-associated*’* infections were those which occurred within or at 48 h of hospital admission, otherwise, they were ‘hospital acquired’ [26]. Clinical isolates were identified using routine laboratory methods [27, 28]. Demographic information (race, age, gender and type of health insurance) and residential addresses were obtained from the EHR. Dates of hospital admission and discharge, patient’s body site(s) of infection, previous *S. aureus* related hospitalizations, and days to a positive culture from time of admission were also collected.

At the neighborhood level, the Census block group where a patient resided was identified [29–31]. We obtained American Community Survey [32] data to determine household crowding, proportion of black by block group, and proportion below poverty. Race/ethnicity, children living in poverty, and median household income were obtained from 2010 Decennial Census [32]. Boundary files for use in GIS for Georgia block groups and counties were downloaded from the National Historical Geographic Information System [33].

#### Race/ethnicity

Race/ethnicity was categorized as white, black, Hispanic and other (‘other’ included: Asian, Native American, multi-racial, Native Hawaiian, and other/declined) and proportions within each block were determined. Based on the proportions of race (black, Asian, American Indian or Alaska Native, Native Hawaiian or other Pacific Islander) reported in the 2010 US Census, we determined which block groups were ‘concentrated’ area of blacks by using the overall percentage of blacks for the state as a reference point. [34] For example, the overall percentage of blacks in Georgia was 30.5% [34] in 2010, so any block group ≥30.5% blacks, was thereby defined as a ‘concentrated’ area of blacks.

#### Poverty

We used the ratio of income to poverty to control for variations in household size and to determine relative poverty by block group. It was determined by dividing the median 2010 family income by the 2010 poverty threshold for a family of four ($22,491) [35] and then assigning block groups as very low income (< 0.99), low income (0.99–1.25), moderate income (1.25–2.50), high income (> 2.5) [36, 37].

#### Household crowding

From US Housing and Urban Development definition, household crowding [38] was any housing unit with more than one person per room [4, 39]. Proportions of crowded households were calculated for each block group.

### Data analysis

The basic unit of observation was each child diagnosed with *S. aureus* infection. Age, gender, and race entered the models as individual level covariates and all others were measured at the Census-block level. All risk factors were categorical except age. Since there were possible unknown correlations between outcomes of interest, and estimates of parameters using a generalized linear model, we applied a generalized estimated equations (GEE) model using an exchangeable covariance structure with model based standard errors; crude odds ratios (OR) were based on conditions determined a priori to be associated with risk of *S. aureus* and used as estimates of relative risks. Adjusted odds ratios (aORs) were calculated using a generalized linear mixed model, allowing for random effects and correlated errors for non-normal data.

Three separate models were applied for multiple regression analyses: Model 1- individual factors only, Model 2 - neighborhood factors, and Model 3- individual and neighborhood factors. Specific individual level and neighborhood level variables were identified, after comparing Model 1 to Model 3, and Model 2 to Model 3; this was done to ascertain which variables remained significant. Variables were included in the final adjusted model if, e.g., when comparing Model 1 to Model 3, the log likelihood statistics for the fitted model were significant for these variables. All tests for significance were two-tailed, and a *p*-value of < 0.05 was considered significant. Statistical analysis was performed using SAS 9.4 (SAS Institute, Cary, NC).

### Spatial analysis

For spatial analyses, we used data at the 2010 US Census block group because of the relatively homogeneous nature and close relationship of smaller census-derived units to health outcomes [40]. Data from all 20 counties of the Atlanta MSA were used for the multilevel and spatial analysis. To improve the geographic visualization of the disease patterns, the maps only show the eight counties that surround the Atlanta City limits; 78% of all cases were in these counties.

#### Geographic distribution of community acquired- MRSA and –MSSA

We measured the risk of CA-MRSA and CA-MSSA as standardized incidence ratios (SIR) which use the observed number of events in an area in the numerator and the expected number of events in the denominator. The number of expected MRSA events was calculated by applying the regional rate of CA-MRSA (14.2 per 100,000) to the number of children in each Census block group and the expected number of CA-MSSA events were calculated by applying the regional rate of CA-MSSA (13.2 per 100,000) to the number of children in each Census block group.

We calculated these SIRs in overlapping units of geography called spatially adaptive filter areas [41] (spatial filters). Spatial filters are aggregations of smaller geographic units, which, by themselves, do not contain sufficiently large enough populations to calculate a reliable disease rate. Spatial filter areas effectively address a common problem in the disease mapping literature called the small number problem [42], which occurs when the population in an administrative unit of geography (i.e., a county or a Census block group) is too small to calculate a reliable disease rate. The sizes of the spatial filter vary across the study area and depend on the number of neighboring Census block groups needed for a threshold value; spatial filters are generally much larger in less populated areas than those in denser areas.

Each spatial filter is centered on a sampling location that we derived from the geometric center of each Census block group within the Atlanta MSA. Here, the smaller units that comprise each spatial filter are the set of Census block groups nearest to each sampling location, which we aggregated together until a threshold value of 30 expected CA-MRSA cases and 30 expected CA-MSSA cases were obtained. To accomplish this, we created a unique spatial filter for CA-MRSA and a unique spatial filter for CA-MSSA and then used whichever produced the largest filter area [43]. We chose 30 because the standard error of the SIR can never exceed 0.183 and any small area with an SIR less than 0.702 or greater than 1.425 will be statistically significant at *p* < 0.01.

The SIRs for CA-MRSA and CA-MSSA were then attributed to the sampling locations and spatially interpolated to reveal a continuous geographic pattern of statistically reliable CA-MRSA and CA-MSSA risk estimates. A by-product of the spatially adaptive filtering procedure is a map of smoothed disease rates, but it differs from other rate smoothing algorithms—such as headbanging [44–46] or localized empirical Bayesian estimation methods [47–49]—which use potentially unreliable disease rates pre-computed in administrative units. The rates calculated in spatially adaptive filters are not pre-computed; we first aggregated the raw number of *S. aureus* cases in neighboring geographic units until a large enough population to calculate a reliable disease rate is obtained and then calculate the disease rates. The effect of this procedure is that all SIRs are based on a minimum level of statistical reliability because the size of the geographic units is based on the principle of choosing an acceptable minimum level of reliability [50].

We also used the same spatial filter areas to calculate covariates. For a given spatial filter area, the numerator for *percent black* is the sum of the number of blacks in the Census block groups that comprise the spatial filter and the denominator is the sum of the people of any race in the same Census block groups. The numerator for the age covariate is the sum of the population younger than 5 years and the denominator is all children younger than 19 years. The numerator for the household crowding is the sum of the number of housing units with more than one person per room and the denominator is the sum of the housing units. We then used Pearson’s R correlation coefficients to measure the association between the percent of *S. aureus* that were CA-MRSA and the three covariates calculated in the spatial filters.

## Results

### Sample size and population characteristics

**Fig. 1:**
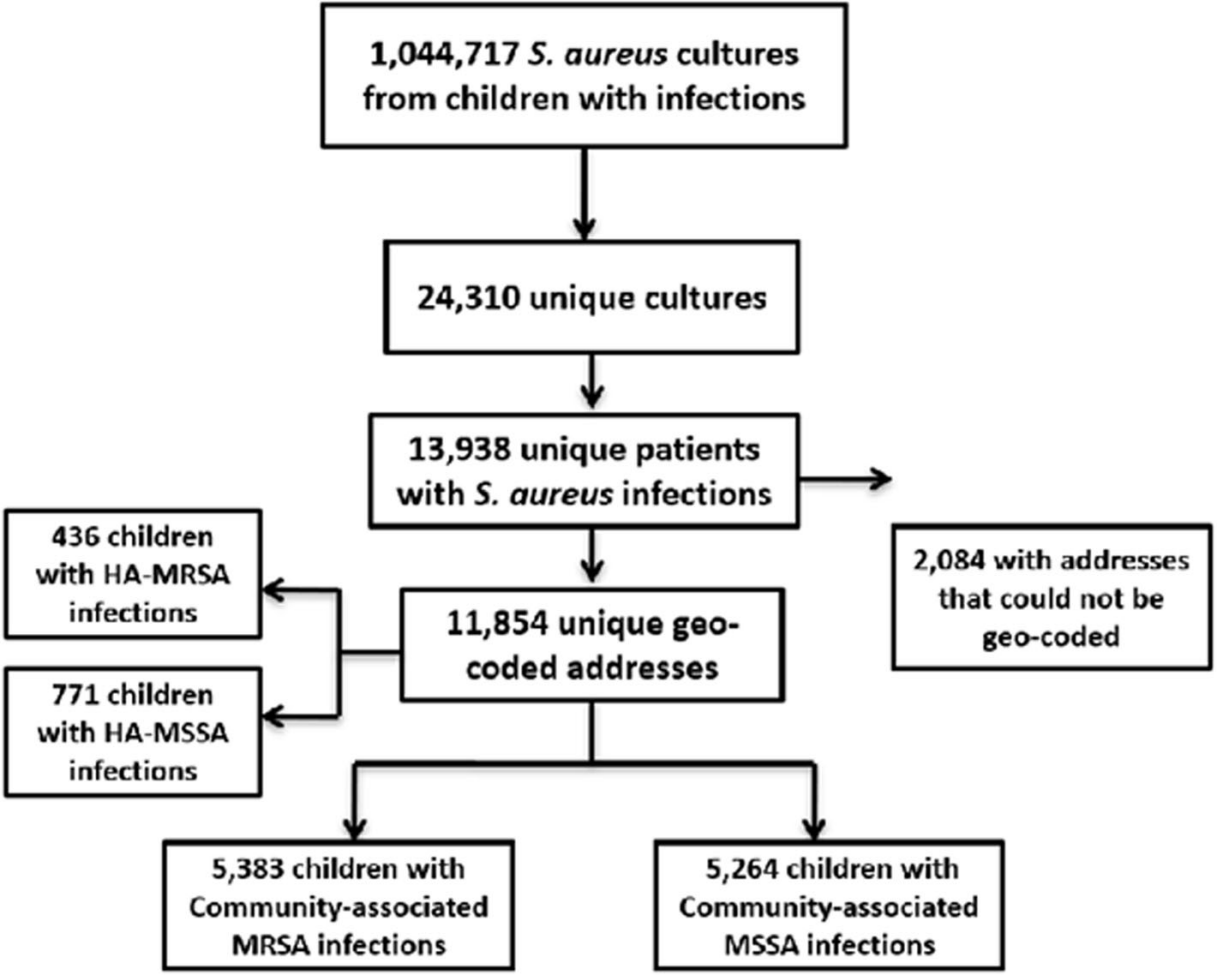
Enrollment Schema. All patients admitted to Scottish Rite Children’s Hospital and Egleston Children’s Hospital from 2002 to 2010 with positive culture for *Staphylococcus aureus (S. aureus)* and had diagnosis of *S.aureus* infection were included. (Data collected did not include any *S. aureus* colonization or carriage information as this was not available.) All unique patients’ addresses were geo-coded and divided into those with community associated methicillin resistant *Staphylococcus aureus* (MRSA) and those with community associated methicillin sensitive *Staphylococcus aureus*

Figure 1 shows the selection criteria for the study. Of 13,938 unique children with a S. aureus infection, 2084 were excluded for invalid addresses (missing, postal office designations, invalid addresses); and 1207 children were excluded with hospital-acquired infections. In 2010, there were 5533 blocks in Georgia and 2097 block groups (38% of all block groups) had one or more observed cases of CA- MRSA. There were a total of 5379 CA- MRSA cases reported during this time period [51]. We determined there were 10,642 community-associated S. aureus infections with complete information, and 50.5% were the antibiotic resistant form, CA-MRSA (Table 1). We then stratified the infections based on the hospital location (Egleston Children’s Hospital or Scottish Rite Children’s Hospital). Differences were found between CA-MRSA and CA-MSSA infections with regard to race, age, gender and type of health insurance. Although white children had the highest proportion of CA-MRSA and CA-MSSA, black children with CA-MRSA was nearly double that of black children with CA-MSSA infections. Children with CA-MRSA also were younger and had higher rates of public health insurance compared to CA-MSSA (*p* < 0.01).

**Table 1:** Patient level and neighborhood characteristics

Variable	Overall (*n* = 10,642)	MRSA (*n* = 5379)	MSSA (*n* = 5263)	*p*-value
Patient Level Characteristics				
Hospital				< 0.001
Egleston	4537 (42.6)	2331 (43.3)	2206 (41.9)	
Scottish Rite	6105 (57.4)	3048 (56.7)	3057 (58.1)	
Race, *n* (%)				< 0.001
Black	3553 (33.4)	2224 (41.3)	1329 (25.3)	
Hispanic	427 (4.0)	212 (3.9)	215 (4.1)	
Other	857 (8.1)	377 (7.0)	480 (9.1)	
Unknown	156 (1.5)	65 (1.2)	91 (1.7)	
White	5649 (53.1)	2501 (46.5)	3148 (59.8)	
Gender, *n* (%)				< 0.001
F	5102 (47.9)	2731 (50.8)	2371 (45.1)	
M	5540 (52.1)	2648 (49.2)	2892 (54.9)	
Age group, *n* (%)				< 0.001
0–3 yrs	4820 (45.3)	2888 (53.7)	1932 (36.7)	
4–12 yrs	3350 (31.5)	1399 (26.0)	1951 (37.1)	
> 12 yrs	2472 (23.2)	1092 (20.3)	1380 (26.2)	
Age, Mean (SD)	6.69 (5.65)	5.95 (5.55)	7.44 (5.66)	< 0.001
Insurance, *n* (%)				< 0.001
Multiple Types	20 (0.2)	10 (0.2)	10 (0.2)	
Private	4876 (45.8)	2101 (39.1)	2775 (52.7)	
Public	5282 (49.6)	3002 (55.8)	2280 (43.3)	
Self Pay	462 (4.3)	266 (4.9)	196 (3.7)	
Neighborhood Level Characteristics				
Household crowding, *n* (%)				< 0.001
≤ 1 person/room	5434 (51.1)	2639 (49.1)	2795 (53.1)	
> 1 person/room	5208 (48.9)	2740 (50.9)	2468 (46.9)	
Black population, *n* (%)				< 0.001
< 40.46%	7322 (68.8)	3361 (62.5)	3961 (75.3)	
≥ 40.46%	3320 (31.2)	2018 (37.5)	1302 (24.7)	
%Below poverty, Mean (SD)	17.1 (14.5)	18.3 (15.1)	15.9 (13.8)	< 0.001

### Prevalence and trend of antibiotic resistant *Staphylococcus aureus* (2002–2010)

**Fig. 2:**
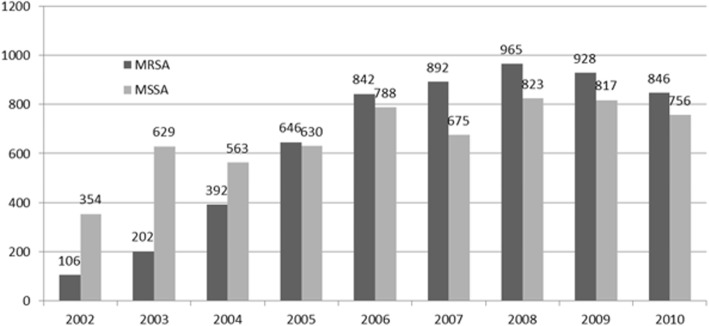
Prevalence of Antibiotic Resistant and Non Antibiotic Resistant *Staphylococcus aureus* Infections in Children, 2002–2010. The number of unique patients with antibiotic resistant (MRSA) and non antibiotic resistant (MSSA) infections from 2002 to 2010 is shown by year. (MRSA, methicillin resistant *Staphylococcus aureus;* MSSA, methicillin sensitive *Staphylococcus aureus*)

CA-MRSA cases increased per year, rising at higher rates compared to CA-MSSA, *p* = 0.01. CA-MRSA infections more than doubled from 23.2% in 2002 to 53.7% in 2006. (Fig. 2).

### Risk factors for community associated MRSA

Children with no or public health insurance had higher odds of CA-MRSA infection compared to those with private insurance (*p* < 0.01). Black children were one and a half times (aOR 1.58 (1.44-1.75, *p* < 0.0001)) as likely as whites to have CA-MRSA infection, after adjusting for gender, age, and health insurance. The youngest category of age (< 4 years) also had increased risk for CA-MRSA (aOR 1.65, 95% CI 1.48, 1.83, *p* < 0.0001). Both crude and adjusted Odds Ratios from Model 1 as described in Methods are summarized in Table 2.

**Table 2:** Individual-Level Factors for CA-MRSA Compared to CA-MSSA Infection for Crude and Adjusted Odds Ratios

Variable	Crude OR (95% CI)	*p*-value	Adjusted OR (95% CI)	*p*-value
Race				
White	Referent	–	Referent	–
Black	1.92 (1.75, 2.10)	<.0001	1.58 (1.44,1.75)	<.0001
Hispanic	1.10 (0.90, 1.35)	0.3359	.81 (.66,1.0)	.0487
Other	0.94 (0.81, 1.09)	0.3989	.73 (.63, .86)	<.0001
Gender				
Male	Referent	–	Referent	–
Female	1.28 (1.18, 1.39)	<.0001	1.22 (1.12,1.33)	<.0001
Age (years)				
> 12 years	Referent	–	Referent	–
4–12 years	.95 (.85, 1.05)	.2725	.95 (.85,1.05)	.3248
< 4 years	1.74 (1.58, 1.92)	<.0001	1.65 (1.48,1.83)	<.0001
Health Insurance				
Private	Referent	–	Referent	–
Self pay	1.70 (1.40,2.08)	<.0001	1.55 (1.27,1.89)	<.0001
Public	1.65 (1.52, 1.79)	<.0001	1.42 (1.30,1.55)	<.0001

Note: The analysis above does not include those patients identified with ‘unknown’ race nor those identified to have multiple types of insurance

At the neighborhood level (Model 2), the unadjusted Model 2 showed significant differences between CA-MRSA and CA-MSSA, with risks for antibiotic resistance being higher in blocks with concentrated black populations (> = 40.46%, *p* < 0.001) and evidence of household crowding (*p* < 0.001). However, household crowding no longer was a significant risk factor for CA-MRSA (*p* = 0.6633), after adjusting for poverty and black concentration, while poverty and concentrated blacks living within blocks remained significant in the adjusted model (*p* < 0.0001 and *p* = 0.0035, respectively). (Table 3) Blocks in the third and fourth quartiles of crowding had higher odds of poverty levels (3rd quartile’s OR 1.32; 95% CI 1.17–1.48; *p*-value < 0.0001; 4th quartile’s OR 1.54, 95% CI 1.38–1.75, *p* < 0.0001). This risk was only slightly higher for CA-MRSA in block groups living below the poverty level (aOR 1.01, 95% CI 1.002, 1.008), compared to aOR of 1.73 (95% CI 1.58, 1.89) if the neighborhood block was concentrated black (*p* < 0.0001).

**Table 3:** Neighborhood-Level Factors for CA-MRSA Compared to CA-MSSA Infection for Crude and Adjusted Odds Ratios

Variable	Crude OR (95% CI)	*p*-value	Adjusted OR (95% CI)	*p*-value
Household Crowding				
≤ 1 person/room	Referent	–	Referent	–
> 1 person/room	1.22 (1.10, 1.34)	<.0001	1.03 (.92,1.14)	.6543
Black Population Concentration				
< 40.46%	Referent	–	Referent	–
≥ 40.46%	2.16 (1.93, 2.41)	<.0001	2.01 (1.79, 2.27)	<.0001
% Below Poverty	1.014 (1.011,1.018)	<.0001	1.006 (1.002, 1.010)	.0033

Multi-level or mixed model analysis (Model 3) showed blacks living in neighborhoods with > = 40.6% blacks, being female, public- or self-pay insurance, and younger age remained highly significant for risk of CA-MRSA infections compared to CA-MSSA, even after adjusting for income, health insurance, gender, crowding, and race/ethnicity (Table 4).

**Table 4:** Multi-factor Model for CA-MRSA Compared to CA-MSSA for Adjusted Odds Ratios

Variable	Adjusted OR (95% CI)	*p*-value
Race		
White	Referent	–
Black	1.68 (1.45, 1.94)	<.0001
Hispanic	.81 (.62, 1.05)	0.1169
Other	.67 (.55, .81)	<.0001
Gender		
Male	Referent	–
Female	1.27 (1.15, 1.41)	<.0001
Age (years)		
> 12 years	Referent	–
4–12 years	.93 (.81, 1.07)	.0260
< 4 years	2.06 (1.79, 2.36)	<.0001
Health Insurance		
Private	Referent	–
Self pay	1.72 (1.33, 2.23)	<.0001
Public	1.51 (1.35, 1.70)	<.0001
Black Concentration		
< 40.46%	Referent	
≥ 40.46%	1.30 (1.13, 1.49)	.0003

### Spatial densities of CA-MRSA and CA-MSSA infections

**Fig. 3:**
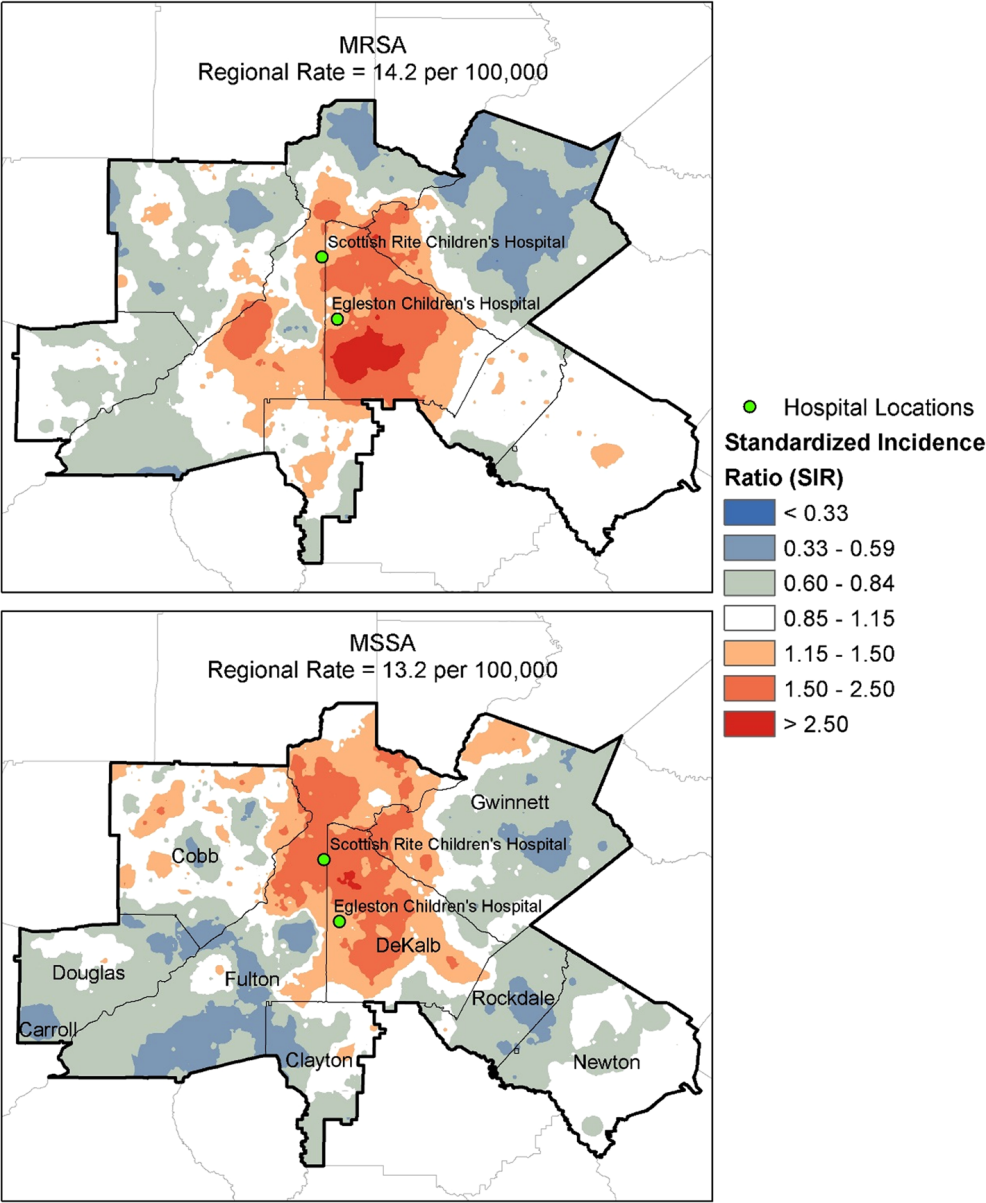
Standardized Incidence Ratios for Community Associated- MRSA and Community Associated-MSSA. The range of standardized incidence ratios are shown for both community associated MRSA and community associated MSSA are displayed across 7 categories with lowest category < 0.33 and highest > 2.5

The spatial distribution of CA-MRSA and CA-MSSA cover similar areas but the SIRs differ in areas affected: CA-MRSA encompasses larger areas with higher SIRs (and a significantly larger area where the ratio is > 2.5). CA-MRSA is much higher than CA-MSSA in the southeastern part of the eight counties that surround the Atlanta city limits. Similarly, this contrast in CA-MRSA and CA-MSSA are seen in southwestern parts of DeKalb county (one of the two counties where the population density is highest for the Atlanta MSA) and specifically, neighborhoods where the rate of ‘overcrowding’ is greater than 50%. In comparison, in areas where CA-MSSA SIRs are highest (northcentral DeKalb county), the rates of CA-MRSA SIR are still relatively high. The highest SIRs are in areas not far from either hospital, where the patients sought care. However, the overall distribution and the shape of the two highest SIRs are quite distinct between those diagnosed with CA-MRSA compared to those with CA-MSSA (Fig. 3).

### Age, race, household crowding spatial relationship to CA-MRSA infections

**Fig. 4:**
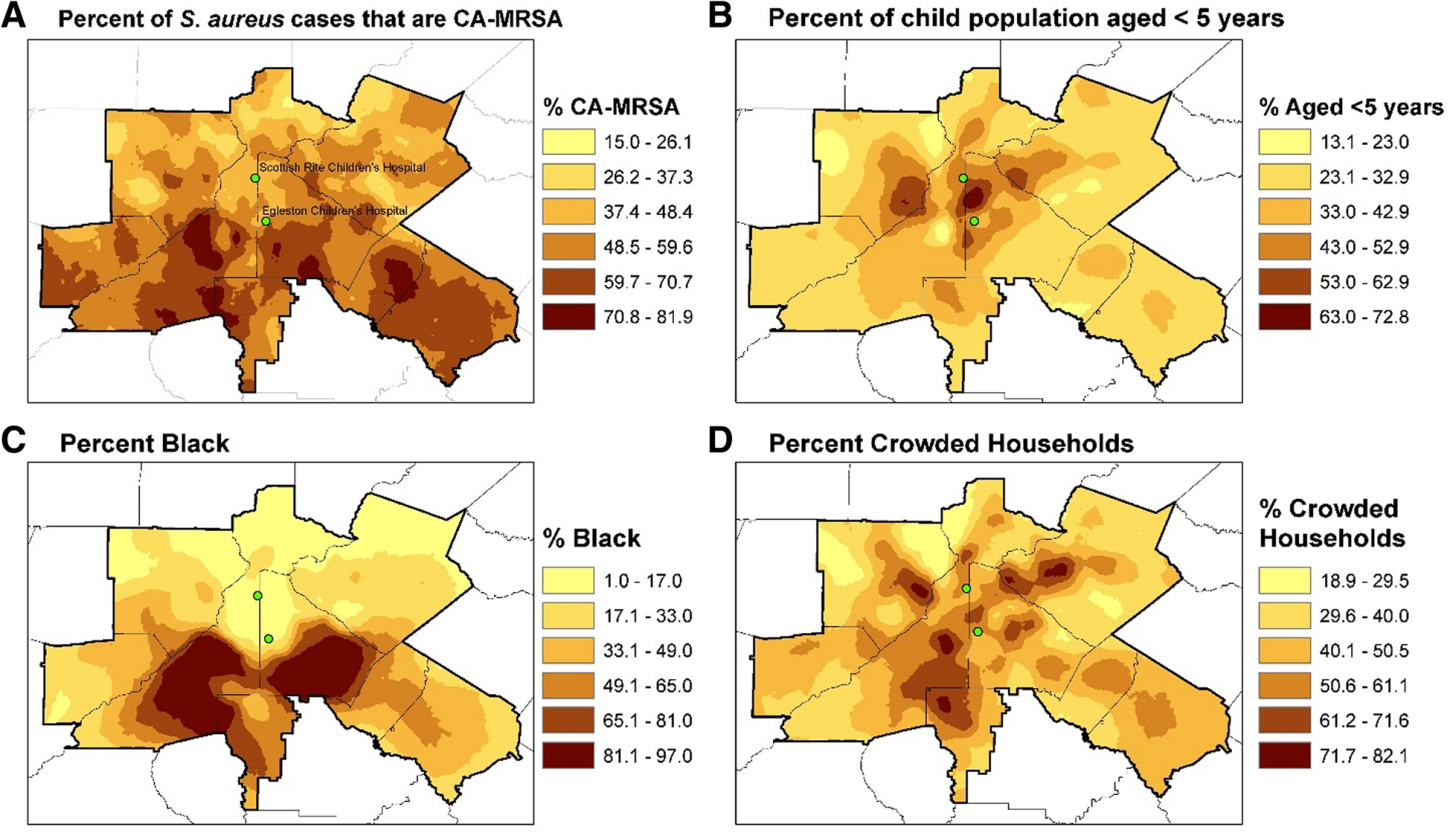
Relationship between Rate of Community-associated MRSA and Race, Young Age, and Household Crowding. Socio demographic population characteristics (percent black, percent crowded households, percent of population < 5 years) are shown for those children who were identified to have community-associated methicillin resistant *Staphylococcus aureus* (MRSA) infections for 2002–2010

We found statistically significant measures of positive association between the spatial filter-level proportion of the *S. aureus* cases that were CA-MRSA and the age (*r* = 0.137, *p* < 0.001), race (*r* = 0.517, *p* < 0.001), and household crowding covariates (*r* = 0.320, *p* < 0.001). These associations indicate that CA-MRSA is elevated in areas where a high proportion of children are younger than age 5 years, where a high proportion of the population are black, and where a high proportion of the households are crowded. These findings are corroborated in our spatial statistical models (Fig. 4). The geographic boundary correlation with race makeup of a geographic area among children with CA-MRSA infections has not been previously reported; previous spatial analyses of CA-MRSA have focused primarily with MRSA colonization of environment or household members, or the hospital’s physical boundaries [52, 53].

## Discussion

Our goal for this spatial analysis was to use electronic health record data to determine which block group-level neighborhoods were most ‘at risk’ for CA-MRSA infections and therefore, would benefit most from targeted primary interventions. We found that the highest proportions of CA-MRSA infections occurred within areas that have the highest levels of household crowding, highest proportion of younger children, and highest proportion of blacks. Although there is some geographic overlap in the CA-MRSA and CA-MSSA densities, the geographic patterns of CA-MRSA were different from that of CA-MSSA even though both types presumably have transmission patterns following that of infectious spatial diffusion. This suggests that the factors driving risk for antibiotic resistant disease (specifically CA-MRSA) may be quite different than those which drive risk for non-antibiotic resistant CA-MSSA. This finding suggest that ‘overcrowding’ is a risk factor for CA-MRSA disease risk.

Fulton and DeKalb counties are very densely populated, and like many core urban areas, have areas with high rates of poverty. ‘Crowding’ and poverty may be factors which mediate antibiotic resistance risk by creating geographically closed communities with more substandard housing conditions and thus, increased opportunities for person-to-person transmission. Over 75% of our CA-MRSA cases came from two of the most densely populated counties in Georgia (Fulton and DeKalb counties). Black residents in these counties also have been described as racially segregated from other populations [54], and this segregation may preclude them from social and economic advantages available to other racial/ethnic groups which are not as segregated.

Our finding of CA-MRSA association with poverty is consistent with other published reports, where higher rates of CA-MRSA infection are identified among urban neighborhoods with higher poverty rates [4]. After adjusting for their individual sociodemographic characteristics, these results show no significant racial differences among the cases (CA-MRSA) and controls (CA-MSSA). This confirms that it is the socio-economic and environmental characteristics of the neighborhood that explain the geographic distribution of MRSA. From a prevention standpoint, providing resources, which otherwise would not exist, to these neighborhoods might be a first step towards meaningful community level intervention.

One strength of this study is the use of a large multi-year dataset of geocoded EHR data to detect spatial patterns in the proportion of CA-MRSA. Another strength is our use of aggregated block group data to create spatially adaptive filters, which results in maps where: 1) all locations have approximately equally reliable estimates of disease risk, and 2) the CA-MRSA and CA-MSSA measures were computed within the same set of spatial filters. We also calculated the covariates using the same spatial filter areas used to estimate the risk of both types of *S. aureus*, which enabled us to measure the association between disease risk and the covariates.

### Limitations

Similar to the limitations associated with using administrative health records [55, 56], the primary limitation of this study is that children who sought care and acquired CA-MRSA from the two hospitals may not be representative of the entire population of children in need of care. For this study, we selected children who accessed care from two pediatric hospitals which represents more than 80% of pediatric admissions in the Atlanta MSA. Although these two hospitals are major healthcare providers for the pediatric population residing within the MSA of Atlanta, this population may not reflect all children infected by *S. aureus* for the geographic region studied. The catchment area of these hospitals may not extend well beyond metropolitan Atlanta counties; and thus, cases closer to other large hospitals may be under reported. Another source of under-reported cases is related to transportation concerns, which may cause some parents to take their child to the nearest hospital rather than a pediatric hospital. Interventions developed would need to factor in the health behaviors characteristic of the populations living in a targeted community.

Another limitation is related to the geospatial correlation analysis of the proportion of *S. aureus* that were CA-MRSA and the covariates. Given spatial dependence inherent in geographic data, which is magnified when using the overlapping spatially adaptive filters, we have violated a central assumption of Pearson’s R correlation that observations be independent. Even though we detected statistically significant associations, the correlation that we measured is higher than what would be observed if the data was not overlapping. Future research using spatially adaptive filters to conduct correlation analysis needs to account for this inflation.

### Future research

The development of targeted community level interventions could be supported by creating community specific reports for the areas with the highest risk of CA-MRSA by characterizing the community according to its unique combination factors known to be associated with antibiotic resistant bacterial transmission. These include neighborhood-level measures of racial composition, household crowding, and age of children as well as information about its built environmental and other social factors. Such a report could be supported using spatial regression modeling to identify where CA-MRSA risk is most associated with these covariates [57]. For example, if types of health insurance and easier access to broad spectrum antibiotics are identified as significantly contributing to CA-MRSA (relative to CA-MSSA) and poor, overcrowded neighborhoods are shown along with their locations of pharmacies and distribution of household income and poverty levels, then at the neighborhood level, we could come up with neighborhood-level (public health) strategies for prevention. Although this study did not address the relationship between CA-MRSA carriage and infection, future studies may include prospectively screening for persistence of carriage among household members and contaminated environmental surfaces associated with patients with recurring infection. Finding ways to decrease bacterial burden on a continuum in these communities may also contribute to an effective prevention strategy. Finally, refining how to spatially ‘weigh’ in socio- and ecological factors, based on geographic place, could combat the spread of antibiotic resistant infections.

## Conclusions

Hospital-based electronic health data systems in conjunction with spatial analyses can provide an effective surveillance system, which could be used to develop strategies for preventing CA-MRSA transmission. This study demonstrates how geocoded EHR data can be used to identify areas of excess risk for *S. aureus* infections which is important for developing interventions to prevent the spread of antibiotic resistant infectious conditions. These methods can be used to identify specific areas to target public health intervention strategies. Such methods have a tremendous potential to prevent the transmission of antibiotic resistant and virulent pathogens in our communities and neighborhoods. While the findings are particular to Atlanta, the methods can be applied globally, where electronically recorded data about *S. aureus* is available.

## Abbreviations

aOR: adjusted odds ratio
CA-MRSA: Community-associated methicillin resistant *Staphylococcus aureus*
ED: Emergency department
GIS: Geographic information systems
MRSA: Methicillin resistant *Staphylococcus aureus*
MSA: Metropolitan Statistical Area
MSSA: Methicillin sensitive *Staphylococcus aureus*
OR: Odds ratio
