# Correction to: Geographic surveillance of community associated MRSA infections in children using electronic health record data

**DOI:** 10.1186/s12879-019-3972-9

**Published:** 2019-05-09

**Authors:** Lilly Cheng Immergluck, Traci Leong, Khusdeep Malhotra, Trisha Chan Parker, Fatima Ali, Robert C. Jerris, George S. Rust

**Affiliations:** 1 Department of Microbiology/Biochemistry/Immunology, Department of Pediatrics and Clinical Research Center, Morehouse School of Medicine, 720 Westview Drive, SW, Atlanta, GA 30310 USA; 2 Children’s Healthcare of Atlanta, 1405 Clifton Road NE, Atlanta, GA 30322 USA; 3 Rollins School of Public Health, Emory University, 1518 Clifton Rd., Atlanta, GA 30322 USA; 4 National Center for Primary Care, Morehouse School of Medicine, 720 Westview Drive, SW, Atlanta, GA 30310 USA; 5 Department of Pathology, Emory University, 1364 Clifton Road Northeast, Atlanta, GA 30322 USA; 6 Florida State University College of Medicine, 1115 W. Call St, Tallahassee, FL 32306 USA

**Correction to: BMC Infect Dis (2019) 19:170**

**https://doi.org/10.1186/s12879-019-3682-3**

Following publication of the original article [1], the authors reported Dr. Kevin Matthews must be removed from the author list.

The authors would like to extend their deep gratitude to Dr. Kevin Matthews for his spatial analyses in calculating the standardized incidence ratios and for the maps created.
